# Electrospun ZnO–SnO_2_ Composite Nanofibers and Enhanced Sensing Properties to SF_6_ Decomposition Byproduct H_2_S

**DOI:** 10.3389/fchem.2018.00540

**Published:** 2018-11-06

**Authors:** Zhaorui Lu, Qu Zhou, Caisheng Wang, Zhijie Wei, Lingna Xu, Yingang Gui

**Affiliations:** ^1^College of Engineering and Technology, Southwest University, Chongqing, China; ^2^Electrical and Computer Engineering Department, Wayne State University, Detroit, MI, United States

**Keywords:** ZnO-SnO_2_ nanofibers, electrospinning, H_2_S, sensing properties, SF_6_ decomposition components

## Abstract

Hydrogen sulfide (H_2_S) is an important decomposition component of sulfur hexafluoride (SF_6_), which has been extensively used in gas-insulated switchgear (GIS) power equipment as insulating and arc-quenching medium. In this work, electrospun ZnO-SnO_2_ composite nanofibers as a promising sensing material for SF_6_ decomposition component H_2_S were proposed and prepared. The crystal structure and morphology of the electrospun ZnO-SnO_2_ samples were investigated by X-ray diffraction (XRD), scanning electron microscopy (SEM) and transmission electron microscopy (TEM), respectively. The composition of the sensitive materials was analyzed by energy dispersive X-ray spectrometers (EDS) and X-ray photoelectron spectroscopy (XPS). Side heated sensors were fabricated with the electrospun ZnO-SnO_2_ nanofibers and the gas sensing behaviors to H_2_S gas were systematically investigated. The proposed ZnO–SnO_2_ composite nanofibers sensor showed lower optimal operating temperature, enhanced sensing response, quick response/recovery time and good long-term stability against H_2_S. The measured optimal operating temperature of the ZnO–SnO_2_ nanofibers sensor to 50 ppm H_2_S gas was about 250°C with a response of 66.23, which was 6 times larger than pure SnO_2_ nanofibers sensor. The detection limit of the fabricated ZnO–SnO_2_ nanofibers sensor toward H_2_S gas can be as low as 0.5 ppm. Finally, a plausible sensing mechanism for the proposed ZnO–SnO_2_ composite nanofibers sensor to H_2_S was also discussed.

## Introduction

Sulfur hexafluoride (SF_6_) insulating gas has excellent insulation performance and arc quenching. It is widely applied in gas-insulated switchgear (GIS) of power system as electrical insulator as well as arc-quenching medium (Beroual and Haddad, 2017; Zhang X. et al., 2017). However, partial discharge and disruptive discharge might occur in GIS equipment during the long run, accounting for the SF_6_ gas decomposing to various decomposition components, such as H_2_S, SO_2_, SOF_2_, SO_2_F_2_ (Tsai, 2007; Liu et al., 2017). Previous researches have reported that these typical decomposition components are able to accelerate the corrosion rate of the GIS equipment and increase the paralysis possibility of the power system (Zhang X. et al., 2016; Li et al., 2017). Therefore, accurate and effective detection of SF_6_ gas decomposition components is significant to estimate and optimize the operation state of GIS power equipment.

Semiconductor metal oxides such as SnO_2_ (Qi et al., 2014; Li et al., 2016; Shahabuddin et al., 2017; Zhou et al., 2018a), ZnO (Zhou et al., 2013; Zuo et al., 2013; Zhu et al., 2018), TiO_2_ (Zeng et al., 2012; Park et al., 2017; Zhang Y. X. et al., 2018), NiO (Zhang Y. et al., 2016; Zhou et al., 2018b,c) are the most investigated group for gas sensors owing to their outstanding gas response and selectivity. Sensing nanostructure with high surface area and full electron depletion is advantageous to enhance the sensing performances (Hao et al., 2012; Miller et al., 2014). In particular, the 1D nanostructures such as nanofibers (Jiang et al., 2016), nanorods (Zhang et al., 2014; Zou et al., 2016), and nanotubes (Kong et al., 2015) have been extensively applied to improve gas sensing properties (Li T. M. et al., 2015; Long et al., 2018). Besides, many studies indicated that the selectivity and other important sensing parameters of semiconductor metal oxide nanomaterials can be enhanced by compositing semiconductor metal oxides (Zhou et al., 2015; Tomer and Duhan, 2016; Wang et al., 2017). Jae-Hun Kim et al. systematically investigated the sensing applications of xSnO_2_-(1-x)Co_3_O_4_ composite nanofibers and reported that the 0.5SnO_2_-0.5Co_3_O_4_ sensor exhibited the most outstanding sensing characteristics (Kim et al., 2017). As one of the most important decomposition components of SF_6_, H_2_S has been widely studied in the past few years. A variety of composite metal oxides like Cu_2_O-SnO_2_ (Cui et al., 2013), CeO-SnO_2_ (Fang et al., 2000), NiO-ZnO (Qu et al., 2016), and PdO-NiO (Balamurugan et al., 2017) have been reported as promising materials for H_2_S gas sensing applications. However, the report of ZnO-SnO_2_ composite nanofibers for H_2_S gas sensing has been only investigated in a limited number of reports.

In this present work, we have successfully synthesized ZnO-SnO_2_ nanofibers by electrospinning method and systematically investigated their sensing performances to H_2_S gas. The prepared ZnO-SnO_2_ nanofibers exhibited significantly improved sensing properties containing high response, low detection limit, low operating temperature and fast response/recovery times to H_2_S gas detection, which can be ascribed to the large surface area of nanofiber structure and the formation of n-n heterojunctions at interface between ZnO and SnO_2_. Finally, a plausible sensing mechanism for the proposed ZnO–SnO_2_ composite nanofibers sensor to H_2_S was also discussed.

## Experimental

### Materials synthesis

Zinc nitrate hexahydrate (Zn(NO_3_)_2_·6H_2_O), stannic chloride pentahydrate (SnCl_4_·5H_2_O), N,N-dimethylformamide (DMF), polyvinylpyrrolidone (PVP, Mw = 1,300,000) and ethanol were of analytical graded and used directly without further purification. All chemicals were purchased from Chongqing Chuandong Chemical Reagent Co., Ltd (Lu et al., 2018).

**Figure 1:**
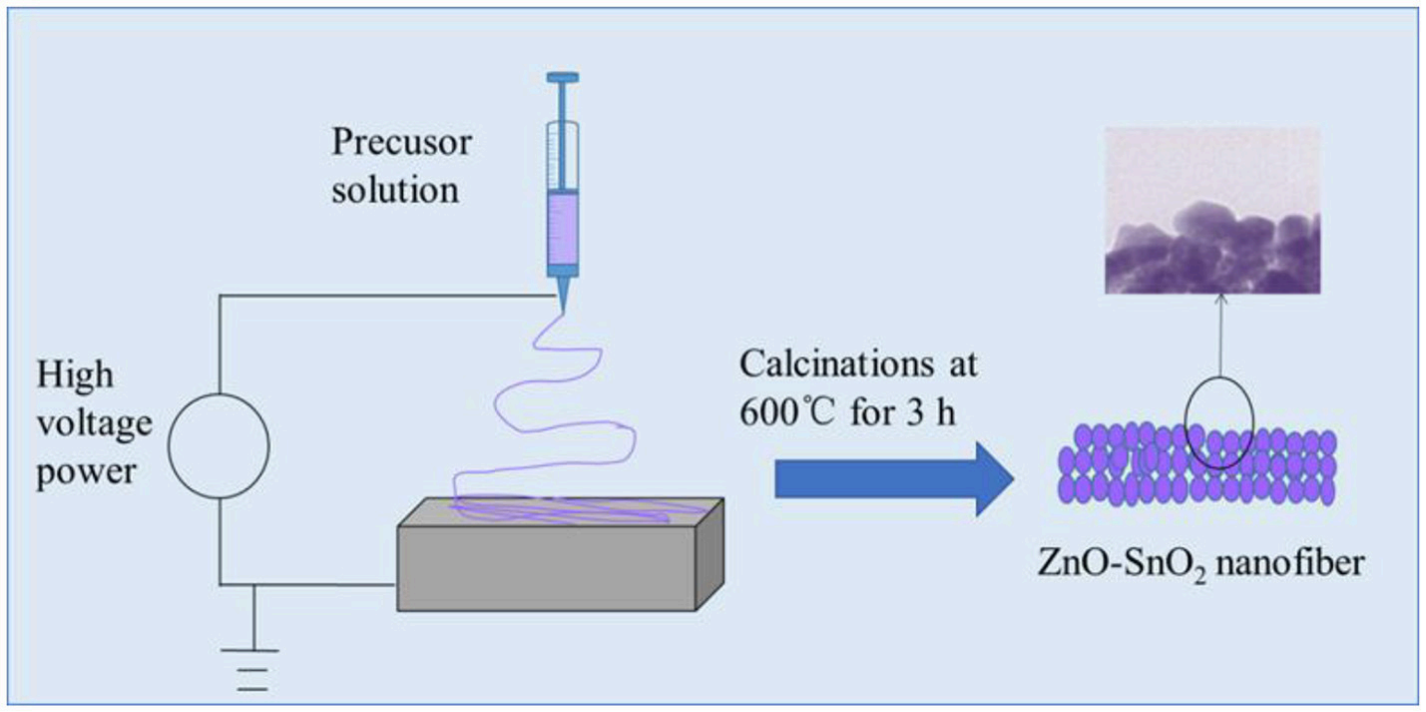
Schematic illustration of producing electrospun ZnO-SnO_2_ nanofibers.

In the typical synthesis of ZnO-SnO_2_ composite nanofibers, 0.7 g of SnCl_4_·5H_2_O and 0.6 g Zn(NO_3_)_2_·6H_2_O (the molar ratio was 1:1) were dissolved in 5 ml of mixed solvents of ethanol and DMF (The volume ratio was 1:1) and stirred for 2 h. Then 2 g of PVP was added to the mixture and stirred for 24 h to form a viscous and homogeneous solution at room temperature. During electrospinning, the obtained mixture was delivered to a glass syringe. A voltage of 25 kV was applied between the flat aluminum foil and syringe at an electrode distance of 15 cm as shown in Figure 1 and the flow rate is 0.7 ml/h. Finally, the electrospun nanofibers were transferred to a tube furnace and the specimens were annealed at 600°C for 3 h in air for the removal of PVP. For comparison, the pure SnO_2_ nanofibers were also synthesized without adding Zn(NO_3_)_2_·6H_2_O. The schematic illustration of producing electrospun ZnO-SnO_2_ composite nanofibers was shown in Figure 1 (Bai et al., 2018).

### Materials characterization

To investigate the structures of electrospun ZnO-SnO_2_ nanofibers, X-ray diffraction (XRD, D/Max-1200X, Rigaku, Japan) analysis was carried out at room temperature using a Rigaku D/Max-1200X diffractometry with Cu-Kα radiation (λ = 1.542 Å) over Bragg angles from 20° to 75° and the scanning speed of 2 deg/min. The morphologies of the electrospun nanofibers were investigated with a field emission scanning electronic microscopy (FESEM, JSM-6700F, JEOL, Japan) and transmission electron microscopy (TEM, JEM-2100, JEOL, Japan) operated at 120 kV. The energy dispersive X-ray spectrum analysis (EDS, Oxford INCA 250, JEOL, Japan) and X-ray photoelectron spectroscopy analysis (XPS, KRATOS X SAM800, Kratos, Kingdom) were tested to analyze the elemental compositions of the sample (Lu et al., 2018).

### Gas sensor measurements

**Figure 2:**
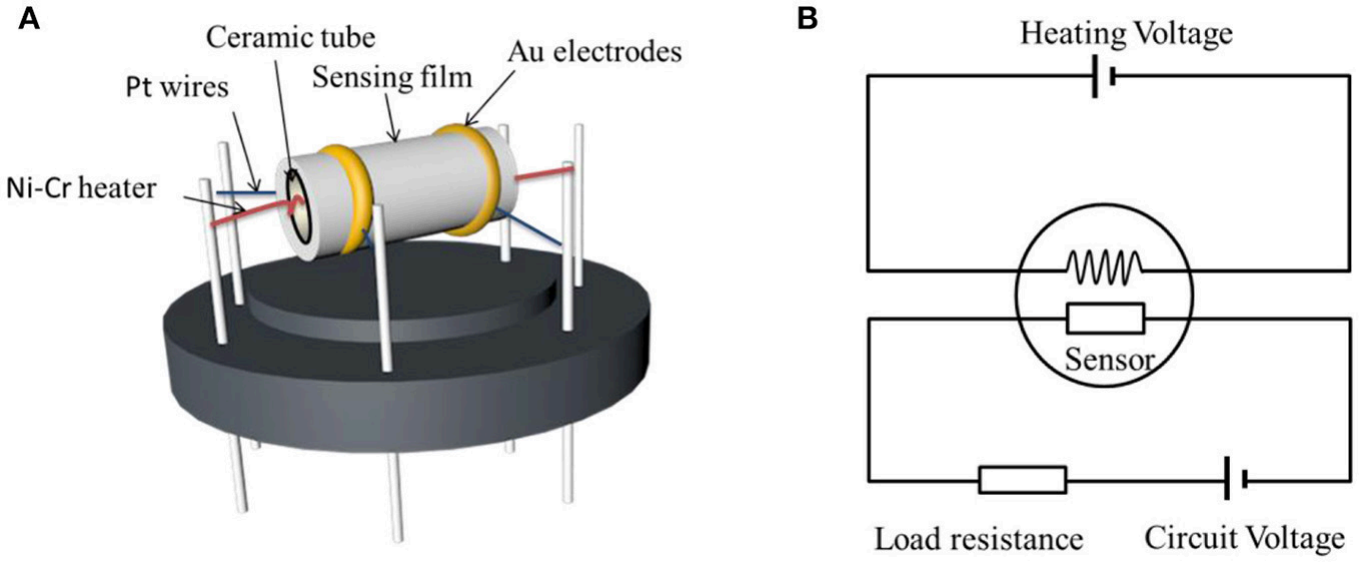
**(A)** Schematic structure of the ZnO-SnO_2_ nanofibers gas sensor and **(B)** theoretic diagram of the test circuit.

Gas sensors were fabricated with a side heated structure as shown in Figure 2A and a theoretic diagram of the test circuit was showed in Figure 2B. As shown in Figure 2A, there are two gold electrodes connected with platinum wire at both ends of the ceramic tube. Firstly, the as-prepared powder was mixed with appropriate amount of anhydrous ethanol and deionized water to form a homogeneous paste (Xu et al., 2015). Then the obtained paste was coated onto a prefabricated ceramic tube to form the sensing film and dried at room temperature for 2 h. Next a Ni–Cr heating wire was inserted into the ceramic tube to finish the side heated H_2_S gas sensor. Finally, the stability of the sensing materials was improved by putting the sensor on the aging instrument of the side heated sensor at 120°C for 10 days. The sensor response was defined as S = R_a_/R_g_ (Zhang Q. Y. et al., 2017), where R_a_ and R_g_ were the resistances of the sensing material measured in air and in atmosphere containing target test gas H_2_S, respectively (Zhu et al., 2017). Gas sensing properties of the obtained sensors were performed with the CGS-8 TP intelligent gas sensing analysis system (Chemical gas sensor-8, Beijing Elite Tech Co., Ltd., China). The response time was defined as the time required by the sensor to reach 90% of the final stable resistance when target gas in. The recovery time is the time required to return to 90% of its original baseline resistance when the sensor was exposed in air again (Nan et al., 2017). The sensing measurement were tested under laboratory condition with room temperature 25°C and constant humidity (50% relative humidity).

## Results and discussion

### Morphology and structure

**Figure 3:**
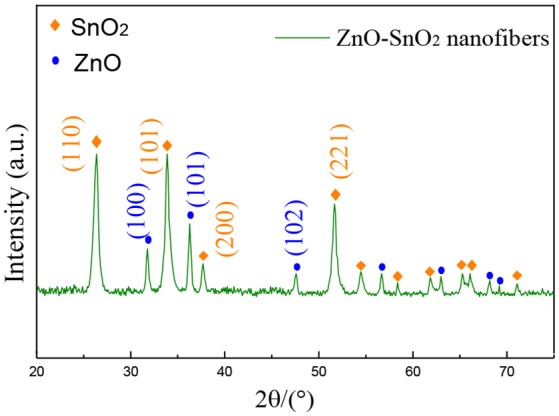
XRD pattern of ZnO-SnO_2_ nanofibers sensing material.

Figure 3 shows the XRD pattern of the electrospun ZnO-SnO_2_ composite nanofibers. It can be seen from Figure 3 that the XRD peaks are in accordance with the hexagonal wurtzite ZnO and tetragonal rutile SnO_2_, compared with the standard pattern of JCPDS card No. 36-1451 and No. 41-1445, respectively. There is neither apparent peak shift nor any other phase corresponding SnO, ZnSnO_3_ and Zn_2_SnO_4_, confirming that there are only SnO_2_ and ZnO co-exist in the prepared material. The result shows a possibility of developing n–n heterojunction at the interface between ZnO and SnO_2_ nanomaterial (Bai et al., 2018). The crystallite sizes of nanoparticels were calculated using Scherrer's equation (D = kλ/βcosθ) (Lu et al., 2018), and the average crystallite sizes of nanofibers were calculated by diffraction peaks (100), (101) for ZnO, and (110), (101), (221) for SnO_2_. After calculation, the average crystallite size of ZnO is 20.1 nm, while it is 19.8 nm of SnO_2_.

**Figure 4:**
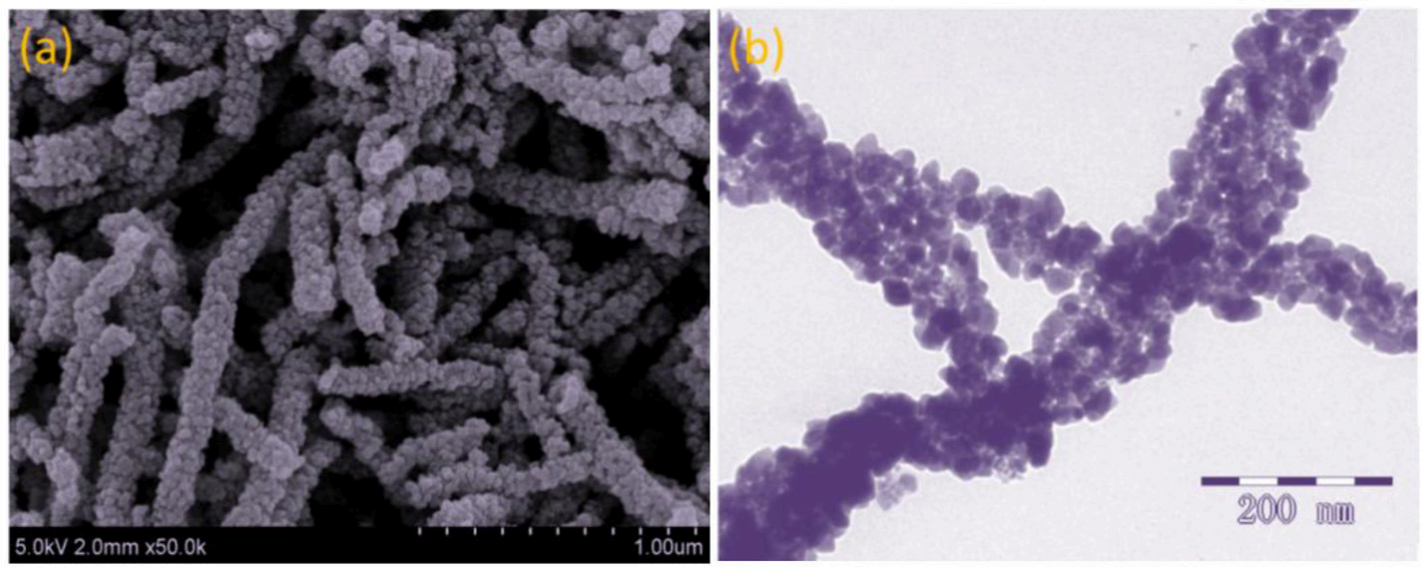
**(a)** FESEM image and **(b)** TEM image of electrospun ZnO-SnO_2_ nanofibers.

Figure 4 presents the FESEM and TEM images of the as-prepared ZnO-SnO_2_ nanofibers after annealing. Figure 4a consists of randomly oriented nanofibers with diameters of 80–150 nm and lengths of 0.5–2 μm. Besides, the ZnO-SnO_2_ nanofibers consist of nanoparticles and surface of nanofibers is rough, which can be attributed to thermal decomposition of PVP caused by annealing. More structural information of the ZnO-SnO_2_ nanofibers is researched by TEM characterization as shown in Figure 4b, the single fiber is composed of grain-like nanoparticles with around 20 nm in size.

**Figure 5:**
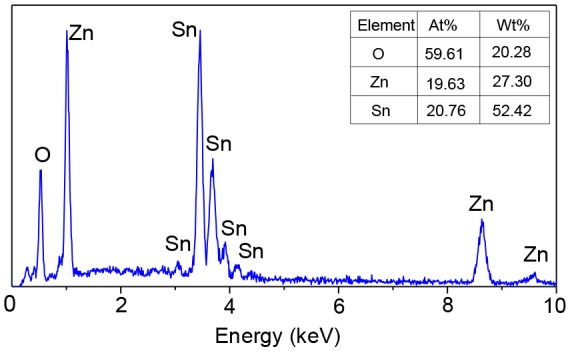
EDS pattern of electrospun ZnO-SnO_2_ nanofibers sample.

Figure 5 demonstrates the EDS spectrum of the electrospun ZnO-SnO_2_ nanofibers. It can be seen that the ZnO–SnO_2_ composite nanofibers are composed of Zn, Sn, and O elements and the atomic ratio of Zn and Sn is about 19.63:20.76.

**Figure 6:**
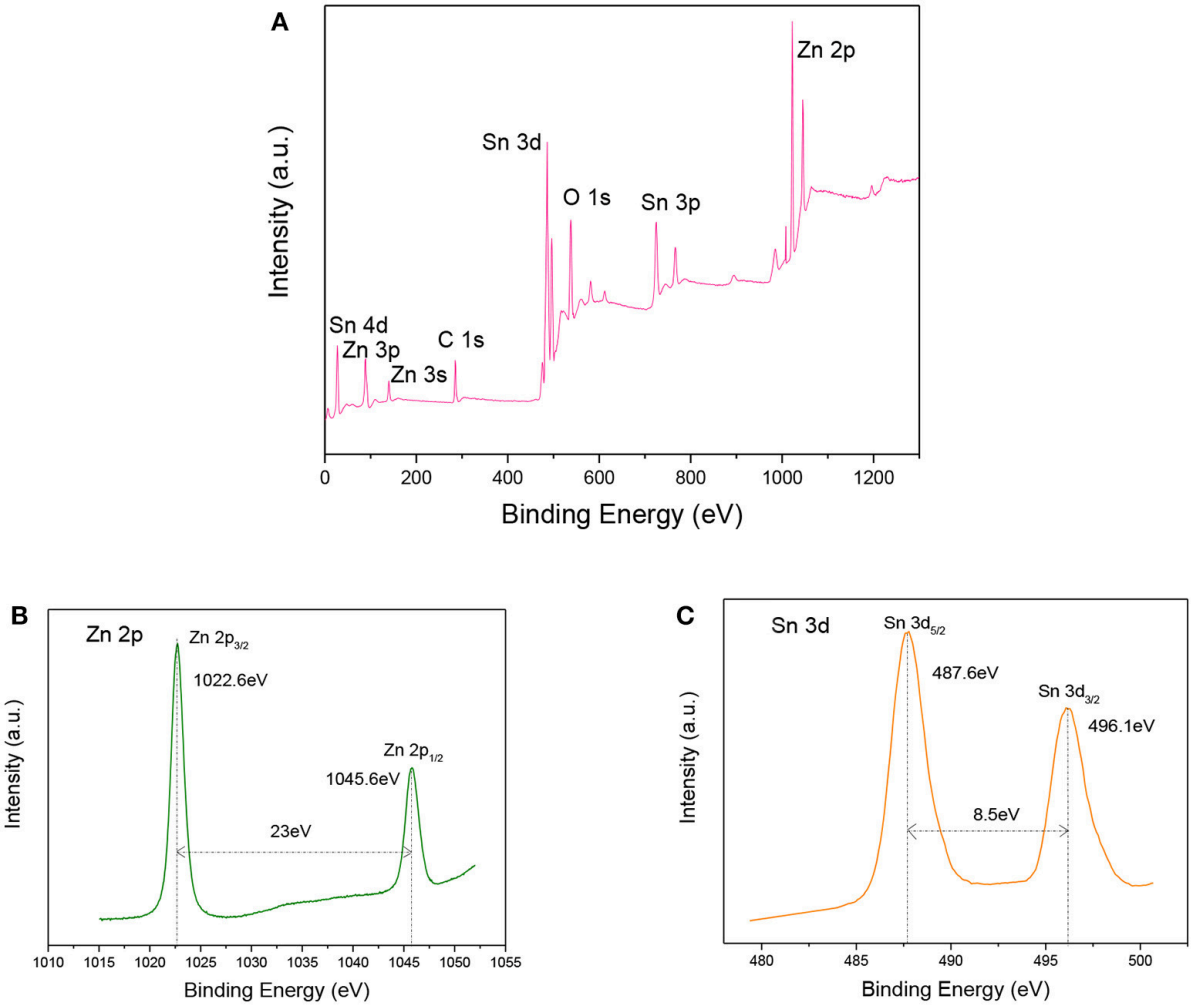
**(A)** Survey, **(B)** Zn 2p, **(C)** Sn 3d XPS spectra of electrospun ZnO–SnO_2_ nanofibers.

To further analyze the compositions and element valences of the ZnO-SnO_2_ nanofibers, XPS tests were investigated. Figure 6A shows the full range XPS survey spectra of the sample. It confirms the presence of Zn, O, Sn from prepared nanomaterial and C element which is due to the carbon contamination. The binding energies were calibrated using C 1 s hydrocarbon peak at 284.54 eV. Figures 6B,C demonstrate the high resolution spectra of the Zn 2p and Sn 3d energy state, respectively. The Zn 2p XPS spectrum (Figure 6B) presents the doublet peaks located at binding energies of 1045.6 eV and 1022.6eV, which corresponds to Zn 2p_1/2_ and Zn 2p_3/2_, respectively (Li W. Q. et al., 2015). The result indicates that the Zn^2+^ is the dominant species in the prepared material and in good agreement with the reported data for ZnO (Zhao et al., 2015). Figure 6C shows the binding energy of Sn 3d_5/2_, Sn 3d_3/2_ are 487.6eV and 496.1eV respectively, which are assigned to the highest oxidation state of Sn^4+^ for SnO_2_ (Hamrouni et al., 2014; Chen et al., 2018). It further confirmed that ZnO and SnO_2_ coexist in the samples.

### Gas-sensing properties

**Figure 7:**
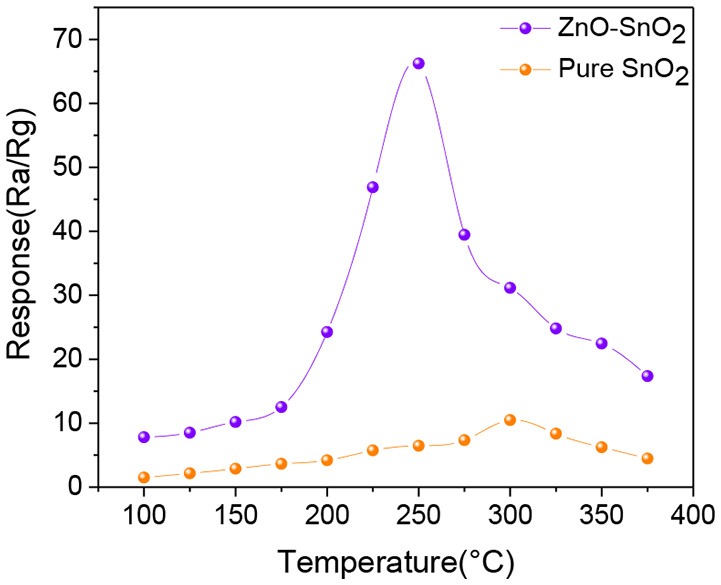
Response to 50 ppm H_2_S at different operating temperature of SnO_2_ and ZnO-SnO_2_ nanofibers sensors.

The gas sensor responses of pure SnO_2_ and ZnO-SnO_2_ nanofibers sensors as a function of temperatures in the range of 100–375°C toward 50 ppm of H_2_S gas were tested and shown in Figure 7. The sensing responses of all the prepared sensors increase with increasing temperature and attain a maximum value at 300 and 250°C for pure SnO_2_ and ZnO-SnO_2_ nanofibers sensor, respectively. With further increase in temperature, the sensing responses begin to decrease because desorption of H_2_S is dominated and the amount of the adsorbed gas onto the surface decreases (Zhang et al., 2018). The response of gas sensor based on ZnO-SnO_2_ nanofibers for 50 ppm H_2_S gas at operating temperature of 250°C is 66.23, while it is 10.49 and 300°C for the pure SnO_2_ nanofibers based sensor. The results indicate that ZnO-SnO_2_ composite nanofibers can obviously improve the response to H_2_S at different working temperatures and reduce the optimal operating temperature.

**Figure 8:**
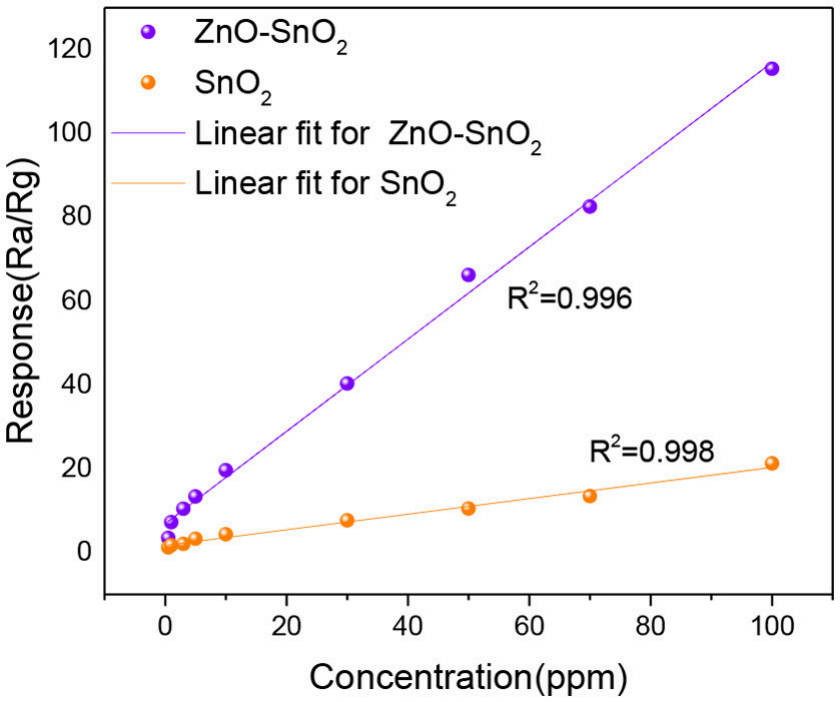
Gas responses for H_2_S gas (0.5–100 ppm) using pure SnO_2_ and ZnO-SnO_2_ nanofibers sensors at their optimal working temperature.

Figure 8 shows the H_2_S gas responses of pure SnO_2_ and ZnO-SnO_2_ nanofibers based sensors to different concentration of H_2_S in the range of 0.5–100 ppm at their optimal operating temperatures measured above. The measured results show that gas responses of the as-prepared gas sensors increase in a good linear relationship from 0.5 to 100 ppm. The linear relationship of the response and gas concentration satisfies linear equation y = 1.1003x+6.90664 for electrospun ZnO-SnO_2_ nanofibers gas sensor. The higher response of ZnO-SnO_2_ nanofibers can be explained by the formation of n-n heterojunctions at the interface between ZnO and SnO_2_. Moreover, the sensor detection limit was defined as the target gas concentration value at which the response is above 3. The response of the ZnO-SnO_2_ nanofibers sensor to 0.5 ppm H_2_S gas can reach up to 3.45, indicating that the detection limit of the sensor for detecting H_2_S gas is as low as sub-ppm level.

**Figure 9:**
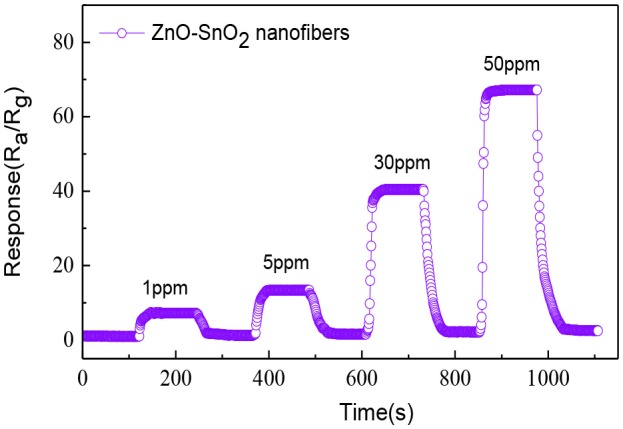
Dynamic response-recovery curve of the sensor based upon ZnO-SnO_2_ nanofibers at 250°C.

The dynamic response and recovery curve of the ZnO-SnO_2_ nanofibers sensor for 1, 5, 30, and 50 ppm H_2_S gas was performed and shown in Figure 9. The obtained sensing response values are about 7.25, 13.37, 40.31, and 66.23, respectively. The ZnO-SnO_2_ nanofibers sensor responds rapidly and could recover to its initial value when it was exposed to air again, implying a satisfying stability and reproducibility of the proposed H_2_S gas sensor.

**Figure 10:**
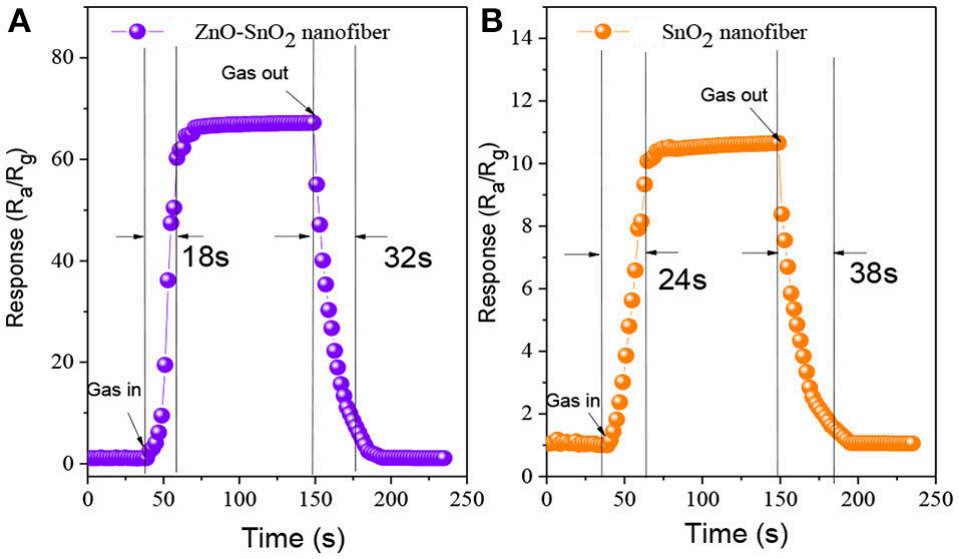
Response-recovery curve of the sensor bases on **(A)** ZnO-SnO_2_ nanofibers **(B)** pure SnO_2_ nanofibers.

Figures 10A,B illustrate the response-recovery curve of electrospun ZnO-SnO_2_ nanofibers sensor and pure SnO_2_ nanofibers sensor to 50 ppm of H_2_S gas at their optimal operating temperature mentioned above. From the curves, it is observed that the response and recovery time of the ZnO-SnO_2_ nanofibers sensor is about 18 and 32 s, respectively, whereas for pure SnO_2_ nanofibers sensor the corresponding values is 24 and 38 s, respectively.

**Figure 11:**
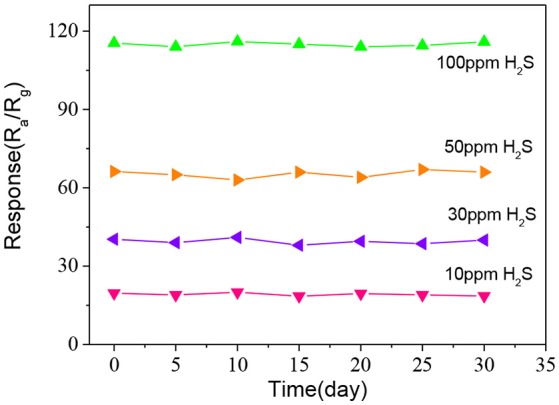
Long term stability property of ZnO-SnO_2_ nanofibers sensor.

Finally, the long-term stability of the fabricated ZnO-SnO_2_ nanofibers sensor was measured to 10, 30, 50, and 100 ppm H_2_S gas at 250°C for 30 days as shown in Figure 11. The measured results show that the response has little change for 30 days and confirm a good stability of the fabricated electrospun ZnO-SnO_2_ nanofibers sensor.

Experimental results of ZnO-SnO_2_ nanofibers sensor have been compared with the results reported by the other workers on H_2_S sensors and presented in Table 1. It can be seen that electrospun ZnO-SnO_2_ nanofibers sensor toward H_2_S can reach a relatively higher response at lower temperature and the response time is relatively shorter than other sensors reported previously. The obtained results indicate that the electrospun ZnO-SnO_2_ nanofibers sensor is promising for H_2_S gas sensing.

**Table 1:** Summary of the H_2_S gas sensing performances of different gas sensor materials.

**Sensor**	**H_2_S (ppm)**	**Temp. (°C)**	**Response (R_a_/R_g_)**	**τ_res_ and τ_rec_**	**References**
Zn_2_SnO_4_ hollow octahedra	50	260	46	10 and 25 s	Ma et al., 2012
V doped In_2_O_3_	50	90	14	15 and 18 s	Liu et al., 2014
Co_3_O_4_-SWCNT composites	100	250	51	>100 s	Moon et al., 2016
Al-ZnO thin film	600	200	30	90 and 209 s	Kolhe et al., 2018
CuO-functionalized WO_3_ nanowires	100	300	79.8	~30 and 20 s	Park et al., 2014
Fe_2_O_3_ -NiO nanoplate	200	200	26	11 and 18 s	Sun et al., 2016
TiO_2_/SiO_2_ composite aerogel film	50	250	13.5	21 and 600 s	Yang et al., 2018
micro/nanostructured In_2_O_3_ porous thin	50	300	30	16 and 30 s	Wang et al., 2016
Gold functionalized MoO_3_ nano flake	15	400	260	60 and 480 s	Munasinghe Arachchige et al., 2018
ZnO-SnO_2_ nanofibers	50	250	66.23	18 and 32 s	This work

*Temp., the optimal working temperature. τ_res_ and τ_rec_ - response time and recovery time*.

### Sensing mechanism

**Figure 12:**
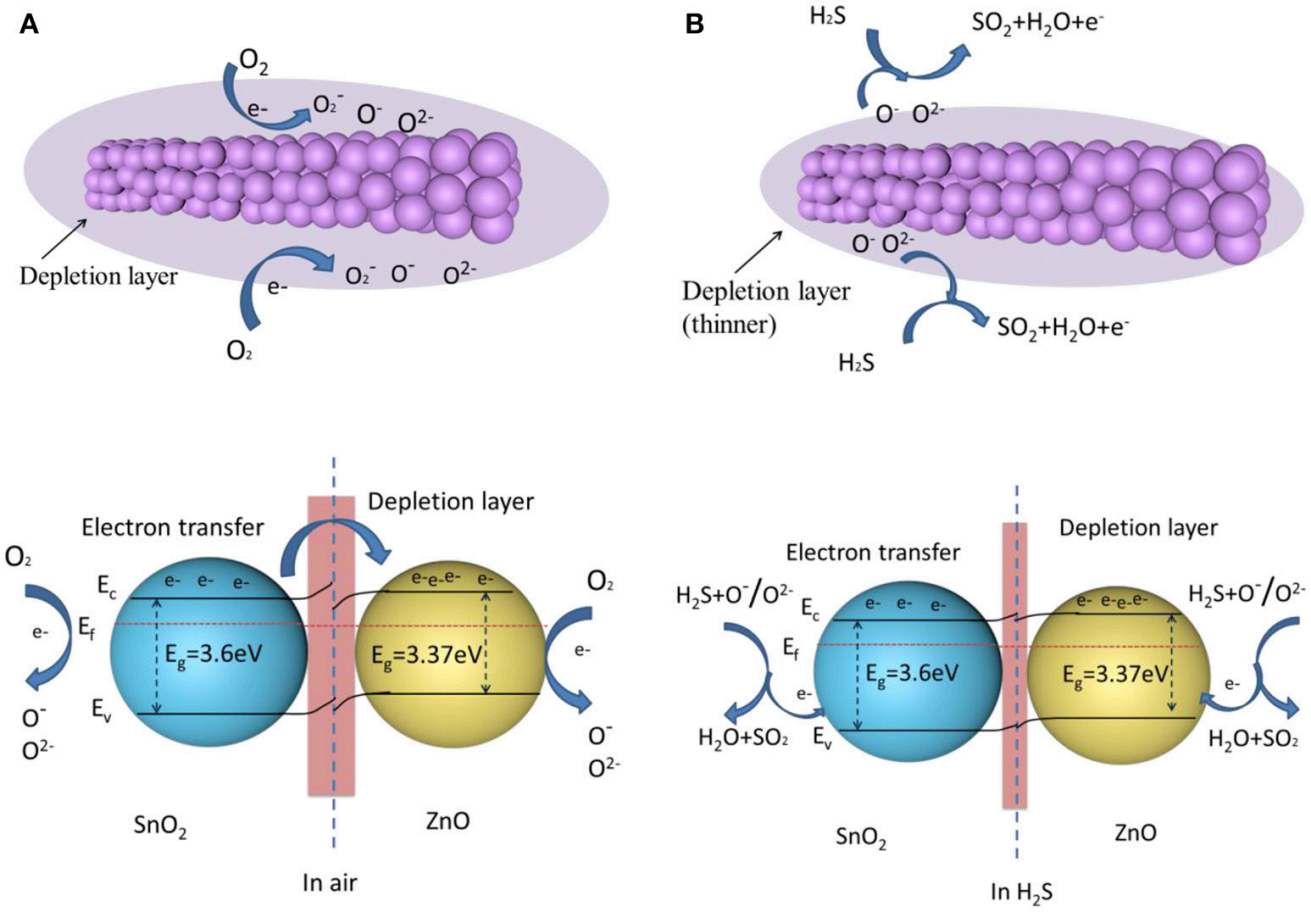
A schematic of the H_2_S sensing mechanism of the ZnO-SnO_2_ nanofibers **(A)** when the gas sensor is exposed in air **(B)** when the gas sensor is exposed in H_2_S (E_f_ is Fermi level; E_c_ is conduction band; E_v_ is valence band; E_g_ is band gaps).

ZnO and SnO_2_ belong to typical n-type semiconductors, characterized by their high free carrier concentration (Hong et al., 2017; Zhou et al., 2018d). The gas sensing mechanism of ZnO-SnO_2_ nanofibers is shown in Figure 12. Due to the sensing mechanism of ZnO-SnO_2_ sensor follows the surface controlled type, the gas sensing properties are ascribed to the change of the surface resistance, which controlled by the adsorption and desorption of oxygen on the surface of sensing materials (Wei et al., 2014). When ZnO-SnO_2_ nanofibers sensor is exposed to air (Figure 12A), the resistance of gas sensor depends on the amount of chemisorbed oxygen species (O^−^, O${}_{2}^{-}$, and O^2−^). The free oxygen molecules are absorbed on the surface and capture electrons from the conduction band of the ZnO-SnO_2_ nanofibers, which causes a depletion layer around the surface and the increasing the resistance (Cheng et al., 2014). When ZnO-SnO_2_ nanofibers sensing materials are exposed to H_2_S (Figure 12B), the target gas reacts with the adsorbed oxygen and then releases the captured electrons into the conduction band of ZnO-SnO_2_ nanifibers to reduce the depletion layer and decrease the resistance.

It is well-known that the chemisorbed oxygen depends on the specific surface area of sensing materials and the operating temperature. ZnO-SnO_2_ nanofibers show a big surface area as shown in Figure 4. It means that adsorption capability of ZnO-SnO_2_ nanofibers was greatly enhanced (Zhang W. et al., 2017). Moreover, O^2−^ and O^−^ species are regarded as the most oxygen adsorption species at 250°C, and the following H_2_S sensing reaction can be considered (Kolhe et al., 2017).

(1) $${\text{H}}_{2}\text{S}+3{\text{O}}^{-}\to {\text{SO}}_{2}+{\text{H}}_{2}\text{O}+3{\text{e}}^{-}$$

(2) $${\text{H}}_{2}\text{S}+3{\text{O}}^{2-}\to {\text{SO}}_{2}+{\text{H}}_{2}\text{O}+6{\text{e}}^{-}$$

In accordance with the definition about gas response (S = R_a_/R_g_), the increasing of R_a_ and decreasing of R_g_ cause that the response of ZnO-SnO_2_ nanofibers is significantly enhanced. Additional electron consumption will occur at the boundaries of ZnO and SnO_2_, which further enhances the gas response. The contact of ZnO and SnO_2_ provides condition for electrons transfer from SnO_2_ to ZnO, which has a higher work function of 5.2 eV compared to SnO_2_ (4.9 eV). It results in the formation of an additional depletion layer in the vicinity region between the ZnO and SnO_2_, eventually generating a potential barrier for electron flow (Choi et al., 2013). The boundary barrier may decrease when the gas sensor is exposed in H_2_S. More electrons of oxygen species transfer to the sensing material by reaction of H_2_S with oxygen species, which results the resistance of the sensing material decreases and the response of the sensor increases. However, pure SnO_2_ does not provide reaction interface, leading to lower gas response. So the ZnO–SnO_2_ composite nanofibers exhibit better sensing properties than the SnO_2_ nanofibers.

## Conclusions

In summary, ZnO-SnO_2_ composite nanofibers were successfully synthesized by electrospinning method and characterized by various techniques. H_2_S sensing properties of the electrospun nanofibers sensor were also investigated. Compared to the pure SnO_2_ nanofiber sensor, the ZnO-SnO_2_ composite nanofibers sensor shows excellent gas sensing response for H_2_S gas, which is attributed to the large specific surface and the heterojunctions between SnO_2_ and ZnO. The proposed ZnO-SnO_2_ composite nanofibers sensor exhibits good linear relationship between sensing response and gas concentration in the range of 0.5~100 ppm and its detection limit is as low as sub-ppm level. Moreover, the proposed sensor achieves good repeatability and long-term stability, making it a promising candidate for detecting H_2_S gas.

## Author contributions

ZL and ZW performed the experiments and analyzed the data with the help from LX and YG. ZL, QZ, and CW wrote and revised the manuscript with input from all authors. All authors read and approved the manuscript.

### Conflict of interest statement

The authors declare that the research was conducted in the absence of any commercial or financial relationships that could be construed as a potential conflict of interest.
